# The Impact of Personalized Exercise Programs on Muscular Functional Capacity and Quality of Daily life

**DOI:** 10.1093/geroni/igaf122.2753

**Published:** 2025-12-31

**Authors:** Semra Ercan

**Affiliations:** Yeditepe University, Istanbul, Istanbul, Turkey

## Abstract

**Objective:**

This study evaluated the impact of a personalized exercise program on muscular functional capacity and daily life activities across various age groups and health statuses.

**Methods:**

A total of 169 participants, aged 16 to 94 with diverse health conditions, were enrolled. incorporating Entrack software and progressive resistive exercise devices by Enraf Nonius, was utilized. Specialized exercise methodology software was also developed. Each participant’s maximum functional capacity across 13 muscle groups was assessed. Four questionnaires evaluated daily life activities and disease severity. Using these data, a personalized exercise regimen was formulated for each muscle group based on a mathematical model. The 12-week program consisted of thrice-weekly sessions, totaling 36 sessions.

**Results:**

Statistical analyses, including T-tests, One-Way ANOVA, Pearson correlation, and Bonferroni multiple comparisons, revealed: • Muscle Functional Capacity: A significant increase was observed (t(169) = 30.58, p < 0.0001), (Cohen’s d = 2.35). • Questionnaire Outcomes: Paired t-tests indicated significant improvements (t(169) = 18.34, p < 0.0001), (Cohen’s d = 1.41). • Correlation Analysis: A significant negative correlation was found between changes in muscle functional capacity and questionnaire scores (r = -0.70, p < 0.0001). Regression analysis yielded R² = 0.49 (p < 0.0001),

**Conclusion:**

Personalized exercise programs may offer potential benefits in reducing psychological and physical stress and the severity of diseases and improving quality of daily life by enhancing muscle functional capacity .This approach is particularly beneficial for older adults who may avoid physical activity due to injury fears or existing health conditions.

